# Estimated Historical and Current Nitrogen Balances for Illinois

**DOI:** 10.1100/tsw.2001.283

**Published:** 2001-10-23

**Authors:** Mark B. David, Gregory F. McIsaac, Todd V. Royer, Robert G. Darmody, Lowell E. Gentry

**Affiliations:** Department of Natural Resources and Environmental Sciences, University of Illinois, Urbana 61801, USA

**Keywords:** soil N, N mineralization, prairies, N_2_ fixation, crop uptake, riverine export of N, denitrification

## Abstract

The Midwest has large riverine exports of nitrogen (N), with the largest flux per unit area to the Mississippi River system coming from Iowa and Illinois. We used historic and current data to estimate N inputs, outputs, and transformations for Illinois where human activity (principally agriculture and associated landscape drainage) have had a dominant impact. Presently, approximately 800,000 Mg of N is added each year as fertilizer and another 420,000 Mg is biologically fixed, primarily by soybean (Glycine max L. Merr.). These annual inputs are greater than exports in grain, which results in surplus N throughout the landscape. Rivers within the state export approximately 50% of this surplus N, mostly as nitrate, and the remainder appears to be denitrified or temporarily incorporated into the soil organic matter pool. The magnitude of N losses for 1880, 1910, 1950, and 1990 are compared. Initial cultivation of the prairies released large quantities of N (approximately 500,000 Mg N year(-1)), and resulted in riverine N transport during the late 19th century that appears to have been on the same order of magnitude as contemporary N losses. Riverine flux was estimated to have been at a minimum in about 1950, due to diminished net mineralization and low fertilizer inputs. Residual fertilizer N from corn (Zea mays L.), biological N fixed by soybean, short-circuiting of soil water through artificial drainage, and decreased cropping-system diversity appear to be the primary sources for current N export.

## INTRODUCTION

Nitrogen (N) is a significant pollutant of surface waters[1] that has been appreciably affected by human activities[2], especially agriculture, which can release or sequester N in soils. N mass balances can be useful to characterize large-scale N dynamics and to assess the relative importance of various N sources and sinks. The fate of anthropogenic N has been studied in considerable detail for the entire U.S.[3], specifically for watersheds draining into the North Atlantic[4]. N inputs included fertilizer, fixation by leguminous crops, atmospheric deposition of oxidized N forms, and the N associated with the import and export of agricultural products. Mass balances for Wisconsin for 1975[5] and Illinois for 1945 through 1998[6], respectively, demonstrated the importance of N fixation and fertilizer as dominant N inputs within the Midwest.

North Atlantic riverine fluxes were found to be about 25% of net anthropogenic N inputs[4]. In the Chesapeake Bay basin, most subwatersheds discharged between 15 and 30% of net annual input[7]. Previous estimated fluxes for Illinois rivers were about 50% of the net anthropogenic inputs, presumably because of the amount of artificial drainage tiles throughout a state with a high percentage of the land in intensive agricultural production[6].

As part of the Committee on Environment and Natural Resources (CENR) assessment of hypoxia in the Gulf of Mexico, all N fluxes in the Mississippi River basin were estimated[8]. In that assessment, inputs and outputs were combined with N fluxes such as mineralization and immobilization of N within the soil organic matter pool. This suggested to some[8] that a large net N mineralization (depletion of soil N pools) was part of the mass balance, whereas others[6] assumed that for current agricultural production (1980 to 1998 in their study), net N mineralization was zero (mineralization equaled immobilization, with no change in soil N pools). McIsaac et al.[9] compared the CENR mass balance[8] to the net anthropogenic input approach, which assumes net N mineralization was zero, and found good agreement of the latter approach with annual Mississippi River N flux. This suggests that in terms of N delivery to the Mississippi River, fertilizer inputs have been more important than net mineralization of soil organic N since 1955.

These N mass balances and relationships to riverine N flux have been used to estimate the role of agricultural practices on N loads in rivers, but most of these studies have focused simply on the period associated with increased fertilizer use (1945 to present). Our objectives were to examine N inputs and outputs and construct N mass balances for Illinois, estimating net anthropogenic N inputs, net mineralization rates, and riverine N fluxes since 1867.

## EXPERIMENTAL METHODS/PROCEDURES

We followed previous approaches for estimating all flux rates[3,6], using data from David and Gentry[6] for 1945 through 1998 and extending their results through 2000. Agricultural statistics were available beginning in 1867, so we used these for Illinois[10] to estimate crop and animal numbers for 1867 to 1944. Where agricultural statistics were not available from the Illinois Department of Agriculture, we used U.S. census information from 1870 through 1920s[11]. We also used U.S. Census of Agriculture information, which is available in 5-year intervals from 1925[12]. Additional information on animal numbers for 1867 though 1907 was taken from Clark[13].

These data sources provided us with (1) annual crop acres and production of corn, soybean, wheat, oat, barley, and hay (both alfalfa and other types); (2) annual numbers of cattle, dairy cows, hogs, chickens, turkeys, horses, mules, and sheep; (3) human population; and (4) N fertilizer sales for the state of Illinois. We used those annual estimates with estimated (1) N crop contents; (2) N fixation rates; and (3) animal intake, manure production, and product N values from David and Gentry[6]. Values for farm horses and mules were taken from various agricultural bulletins[14,15,16]. Estimates of atmospheric N deposition followed David and Gentry[6], with further scaling of inputs back to 1900 using U.S. Environmental Protection Agency estimates of national emissions[17]. Anthropogenic deposition of N prior to 1900 was assumed to be 50% of the 1900 value. We treated ammonia emissions as being redeposited within the state, and therefore did not estimate either flux[6].

Land-use data were taken from agricultural statistics or U.S. Bureau of Census records. From these data we estimated (1) the cumulative land area that had been tilled each year since 1850, (2) amount of land remaining in forest or prairie, and (3) area of land in pastures. These records were available for 10-year intervals in the 19$^{th}$ century and, generally, for 5-year intervals during the 20$^{th}$ century. We used linear interpolation to estimate annual values between census years. For land in prairies, we used an N fixation rate[18] of 8 kg N ha$^{-1}$ year$^{-1}$, whereas we followed David and Gentry[6] in assigning N fixation rates of 218, 116, and 15 kg N ha$^{-1}$ year$^{-1}$ for alfalfa (*Medicago sativa* L.), other hay, and pasture, respectively.

Net mineralization of N from soil organic matter was estimated by calculating the land area plowed for the first time on a 10-year interval, assigning it to either prairie (Mollisol) or forest (Alfisol), and estimating the total N content in the upper 15 cm and the annual release of N. This approach suggested that 8.5 million ha of prairie and 2.4 million ha of forest were plowed by 1915, with most of the area plowed by 1875. To estimate total N in the soils, we used spatially weighted average soil organic matter values for Illinois circa 1980[19], converted soil organic matter to C by dividing by 1.724, and then calculated total N assuming a C:N ratio of 12. These 1980 total N percentage estimates were 0.21 and 0.09 for Mollisols and Alfisols, respectively. We then estimated the average N content prior to cultivation assuming either a 42[20] or 33% loss[21], and a bulk density increase from 1.1 to 1.3 Mg m$^3$ following cultivation. The 0- to 15-cm presettlement soil N contents were thereby estimated as 6.1 to 7.1 Mg N ha$^{-1}$ for Mollisols, and 2.6 to 3.0 Mg N ha$^{-1}$ for Alfisols. Following Jenny[20], we estimated that N (high estimate) was mineralized at the rate of 25% of the original total N for the first 20 years following cultivation, 10% in the second 20 years, and 7% in the third 20 years. For a low estimate, we used a total of 33% loss based on Mann's[21] meta-analysis and assumed this loss occurred at the rate of 20, 8, and 5% of soil total N for the three 20-year periods following cultivation. Finally, we assumed that organic N in the 15- to 30-cm depth of soil was 70% of that in the 0- to 15-cm depth, and that 10% was lost[21]. These estimates were necessary because the first soil N measurements (or C) in Illinois were from 1901 to 1905[22], for soils that probably had been under cultivation from anywhere from a few years to as much as 70 years.

Early studies reported N contents for virgin prairies in states to the west and northwest of Illinois[23,24,25,26], with values in the range of our estimates, but often with a large amount of variation. Clearly, N concentrations in presettlement prairie soil varied widely due to differences in inherent productivity, texture, and drainage. The available data do not allow a precise assessment of average soil N concentrations or content of Illinois soils during the presettlement period. Our analysis of the available data and literature indicated that the average soil N content in the surface 15 cm of Illinois Mollisols prior to cultivation was probably between 6 and 8 Mg N ha$^{-1}$. Northern sites with poor drainage would have had N contents greater than these values, whereas upland and southern Mollisols would have had lower N contents.

Finally, to estimate average N flux in rivers leaving the state of Illinois we multiplied the available N (sum of net anthropogenic N inputs and net mineralization) values by a yield or transfer percentage. We estimated that 20% of the available N before 1880 was exported in the rivers of the state, similar to the value obtained by Howarth et al.[4] for a wide range of watersheds. Tile drainage appears to increase the percentage of N that is transported to rivers, and after 1878 the use of tile drainage expanded rapidly because Illinois changed its constitution and gave drainage districts greater authority to levy taxes and appropriate land[27]. Unfortunately, there are no consistently collected statistics on the effectiveness or intensity of drain tile installation over time. To estimate the changing effects of tile drainage on available N transport to rivers, we used yield percentage values of 25% for 1881 to 1890, 35% for 1891 to 1920, 40% for 1921 to 1945, 45% for 1946 to 1970, and 50% since 1970 (the value determined by David and Gentry[6]).

## RESULTS AND DISCUSSION

The Illinois statewide N budget has gone through many changes during the last 140 years, with inputs coming predominantly from $N_2$ fixation by prairies, hay production, and pastures until the 1930s, and then changing to fertilizer and soybean $N_2$ fixation (Fig. 1). Both the source of N inputs and magnitude have changed. N inputs increased from about 200,000 Mg year$^{-1}$ before 1930 to about 1,200,000 Mg year$^{-1}$ today. The other large change has been N in harvested grain, which has also increased greatly since the 1940s with the introduction of fertilizer, hybrid varieties, and new crops (soybean). During the same period, animal numbers and grain consumption have decreased, whereas human consumption of N in food has slowly increased.

Combining inputs and outputs allows calculation of N that could be transferred to surface waters. Until 1920 the amount was approximately 120,000 Mg N year$^{-1}$ (Fig. 2). It then increased to nearly 200,000 Mg year$^{-1}$ in the 1930s, decreased in the 1950s as the prairie soils became depleted in N, and greatly increased with widespread fertilizer use in the 1980s and 1990s to about 500,000 Mg year$^{-1}$. For the 2000 crop year we estimated a net input of 198,000 Mg, which is the lowest amount observed since 1963. This was due to a large crop harvest, particularly of soybean, which has a high N content. The recent leveling off and perhaps decrease in net N inputs is likely different than world trends due to stable fertilizer use and increasing crop production in Illinois vs. rapidly increasing fertilizer applications in much of the developing world[28].

In the previous Illinois N mass balance[6], soil N mineralization was assumed to be balanced by immobilization since 1978, with no net change in soil N pools. It is possible that recent adoption of no-till production practices has increased N immobilization (along with C), although this would apply primarily to the portion of the acres under continuous no-till. Crop residue surveys[29] in Illinois indicate that only 10 to 20% of the corn acres are planted using no-till and a portion of these are likely to be managed as short-term no-till. Additionally, although several studies have shown that soil organic matter in the surface layers had increased after the adoption of conservation tillage, the few studies that have examined changes below 20 cm have reported a significant number of locations where the increased organic matter in the surface horizon was offset by reduction in organic matter

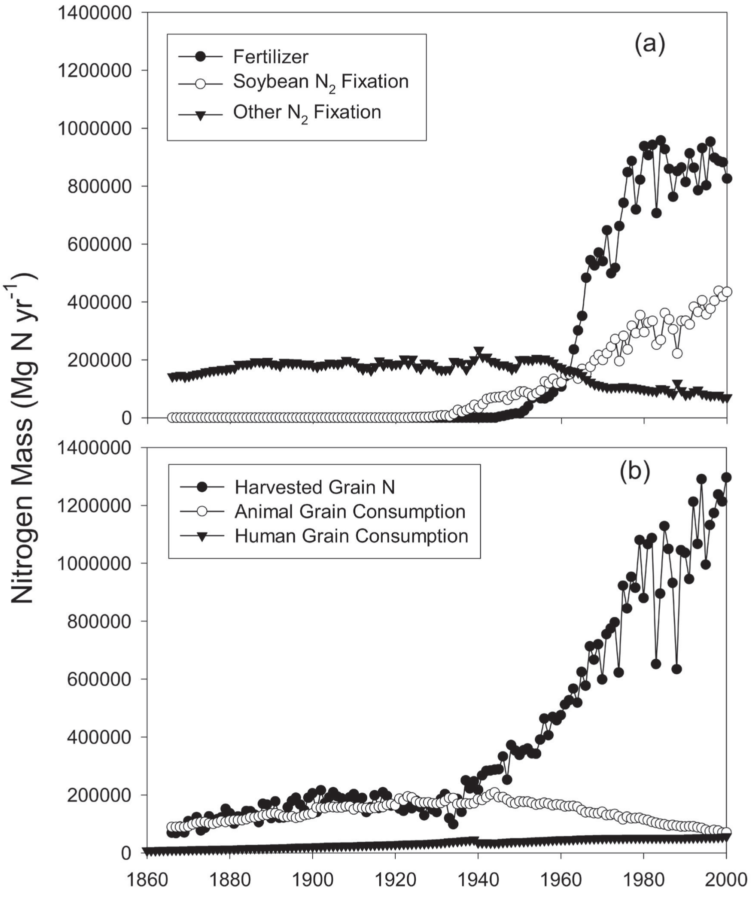

**FIGURE 1.** Annual N (**a**) inputs from fertilizer N, soybean $N_2$ fixation, and other $N_2$ fixation (alfalfa, other hay, pastures, and prairie) and (**b**) harvested grain N (sum of corn, soybean, wheat, and oat), animal grain N consumption (sum of sheep, cattle, dairy cows, hogs, chickens, turkeys, and horses), and human grain consumption in Illinois.

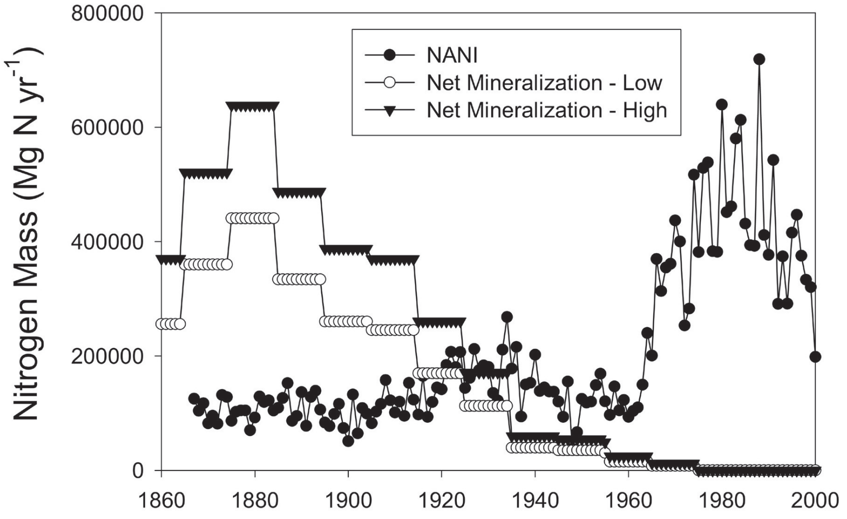

**FIGURE 2.** Net anthropogenic N inputs (NANI), along with low and high estimates of net mineralization of soil organic N following initial plowing of prairies and forested soils in Illinois.

deeper in the soil profile[30,31,32]. Therefore, the influence of increased use of no-till on soil immobilization statewide is likely to be small.

We have estimated the release of N from soil organic matter for 60 years following the initial plowing of the landscape (both low and high estimates) (Fig. 2). N mineralization appears to have peaked around 1880 between 450,000 and 650,000 Mg year$^{-1}$. This input alone is on the same order of magnitude as net anthropogenic inputs during the 1980s and 1990s. The sum of net anthropogenic N inputs and soil N mineralization reached a minimum around 1950 (before widespread use of fertilizer N), but then increased dramatically after 1960 as inorganic N fertilizers became widely used. Between 1867 and 2000, there have been two major sources of N in the Illinois landscape beyond that needed for crop production: (1) release of mineralized N stored by plant production and $N_2$ fixation in prairie and forest soils during the 11,000 to 12,000 years following loess deposition[33]; and (2) N fertilizer. Both of these sources were similar in magnitude.

Comparing the sum of net inputs plus N mobilized from soil (an index of availability of N in the soil for leaching) averaged for 10-year periods provides an overview of changes in the Illinois N balance (Fig. 3). The largest N availability was estimated to be around 1880, primarily from net mineralization. N availability declined from 1880 to 1950 even though net inputs increased slightly over this period, because N mineralization from soils declined dramatically. By 1990, net mineralization had become insignificant yet N availability increased because of increases in net anthropogenic inputs. This increase was due primarily to the use of fertilizer.

Based on our assumptions of transport of available N from land to water, riverine export of N was estimated to have been about 145,000 Mg year$^{-1}$ in the 1880 and 1910 periods. Although N mineralized from soils declined dramatically between 1880 and 1910, net inputs and riverine transport remained relatively constant. We believe this was a result of expansive tile drainage that increased the rate of N movement from fields to streams and reduced both the extent and duration of saturated soils, thus reducing soil denitrification. By 1950, riverine export was probably at a minimum (72,000 Mg year$^{-1}$) for the period of examination, with low mineralization and net anthropogenic N inputs. Riverine export then increased to 210,000 Mg year$^{-1}$ by the 10-year period centered on 1990. Using river flow and measured N concentrations, an estimate of riverine export of 242,000 Mg year$^{-1}$ was made for the 1986 to 1995 period[6]. Our riverine export estimates are presented as average values due to the lack of available data to examine a year-by-year loss. It must be noted that exports in a given year could have been — and likely were — quite different than the average, due to differences in precipitation (drought years vs. greatly above-average precipitation).

We constructed mass balances of N for the state for 10-year periods with mid-points of 1880, 1910, 1950, and 1990, using the average of low and high mineralization estimates (and therefore the average low and high riverine export). All N was accounted for by assuming that available N from net anthropogenic N inputs and mineralization that was not exported in rivers was denitrified either in fields, wetlands, or during riverine transport. The 1880 mass balance was dominated by net mineralization of soil N, with a corresponding large denitrification flux (Fig. 4). The mass balance indicated that grain N exports were essentially balanced by the food and feed imports to the state necessary to meet the demands of the resident animals and humans. Animals consumed large amounts of N, beyond that provided by Illinois grain from $N_2$ fixed in pastures and in hay fields (flux not shown). Humans could also consume significant quantities of fin and shellfish from Lake Michigan. Deposition was low, and sewage was at most a small percentage of riverine export.

There was still no net export of grain N by 1910, but net mineralization and denitrification were estimated to have been about 45% less than in 1880. An estimate of the N flux in the Illinois River, which drains about half of Illinois, was 33,000 Mg year$^{-1}$ between 1897 and 1902[34]. If we double that value, as a statewide estimate, it is much less than our riverine flux of 146,000 Mg N year$^{-1}$ calculated for the same time period. If we have over-

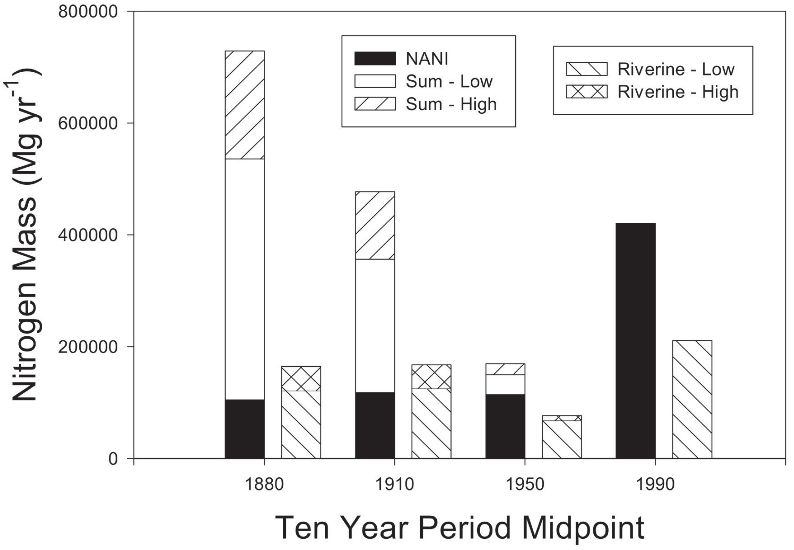

**FIGURE 3.** Sum of net anthropogenic N inputs (NANI) and net soil N mineralization estimates (low and high values) for 10-year periods, along with riverine N export estimates using low and high mineralization values and estimated transport coefficients for Illinois.

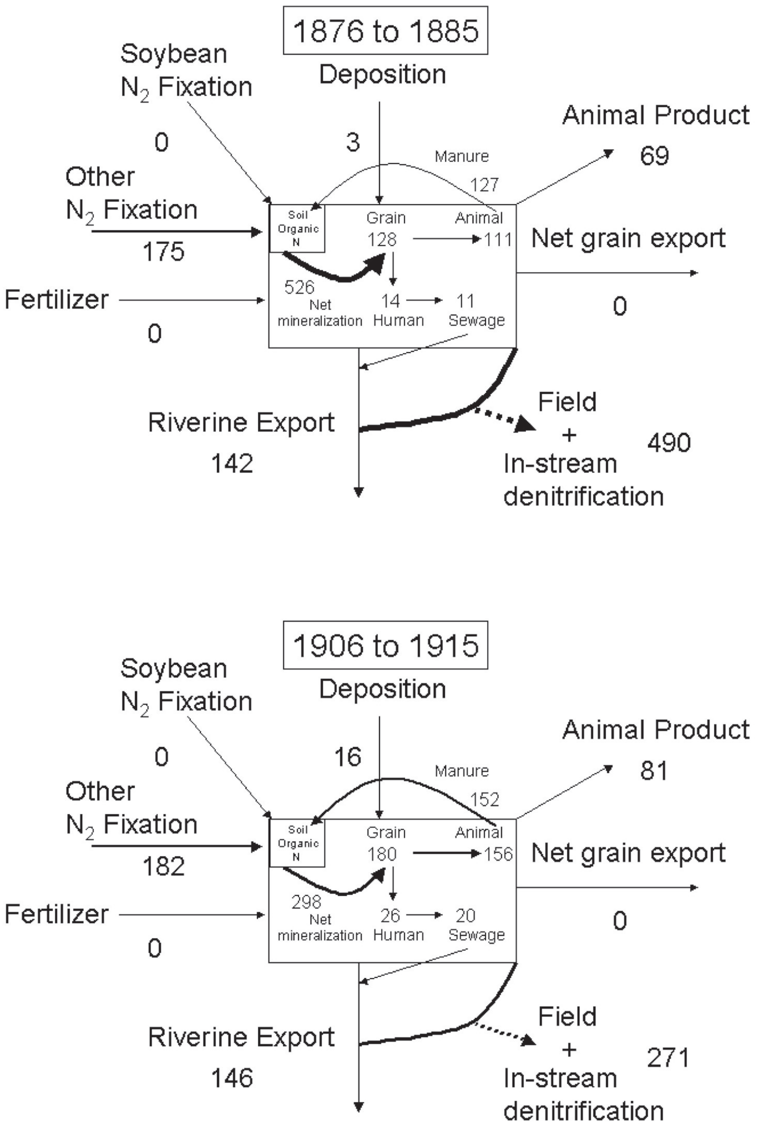

**FIGURE 4.** N mass balances for Illinois, showing average fluxes for 10-year periods with midpoints of 1880 and 1910. Mean of low and high net mineralization and riverine export flux values was used. Not all components included in the balance are shown. Lines are drawn proportional to flux rate. Units are 1000 Mg year$^{-1}$.

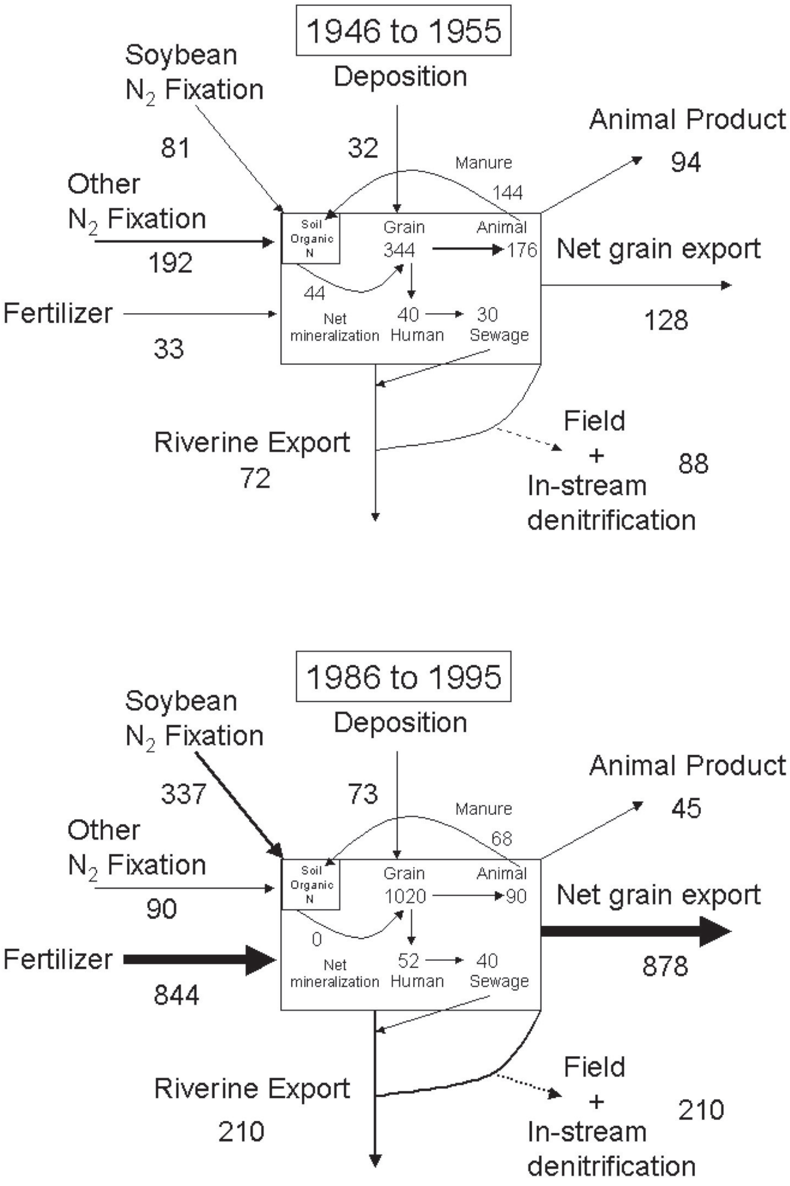

**FIGURE 5.** N mass balances for Illinois, showing average fluxes for 10-year periods with midpoints of 1950 and 1990. Mean of low and high net mineralization and riverine export flux values was used. Not all components included in the balance are shown. Lines are drawn proportional to flux rate. Units are 1000 Mg year$^{-1}$.

estimated riverine transport it is likely we have underestimated the amount of N denitrified in the rivers of Illinois. At this point in history streams and rivers received untreated domestic sewage and livestock waste. This loading of organic matter created large regions of anoxia[35] that would have facilitated denitrification in both the water column and the river sediments. The importance of riverine denitrification probably peaked around the late 19$^{th}$ and early 20$^{th}$ centuries. Additionally, there is considerable uncertainty in our estimates of N transport efficiency and N mineralization rates, especially for the period prior to 1950. Consequently, the magnitude and timing of these estimates should be considered as approximate, albeit based on the best available data and current understanding of N dynamics.

By 1950, Illinois was a net exporter of N in the form of food and feed grain and no single component dominated the balance as it had in the 1880 and 1910 periods, or would by 1990 (Fig. 5). Riverine export and denitrification were presumably at minimums for this period of the study. By 1950, most larger streams and rivers in Illinois had been dredged and channelized, thereby reducing the residence time of the water and disconnecting the rivers from their floodplains — an impact that affects nutrient processing[36]. Also, sewage treatment had become widespread by 1950 and this increased the oxygen concentration of streams and rivers. Oxygenation, combined with channelization and dredging, appears to have acted to reduce the role of riverine denitrification. In the 1990 mass balance, fertilizer and soybean $N_2$ fixation dominated, along with net grain export of N. Riverine export was the highest estimated since intensive agricultural production began. The large amount of riverine transport of N under present conditions in Illinois results from a combination of factors that have arisen from the agricultural development of the state. Tile drainage effectively moves fertilizer N from cropland to receiving streams, particularly following precipitation events[37]. Tile drainage, dredging, and channelization have altered hydrological patterns of movement and retention of surface water within Illinois and created conditions generally unfavorable for denitrification, although isolated "hot spots" probably exist (e.g., reservoirs, remaining/restored wetlands). We suggest that the widespread application of N fertilizer, the efficiency of tile drainage, and the altered hydrology of streams and rivers collectively explain the current export of riverine N from Illinois and probably the agricultural Midwest in general.

## CONCLUSIONS

The mass balance of N in Illinois has been affected by two large sources during the period of European settlement and intensive agricultural production: (1) release of stored soil organic N prior to 1950 and (2) use of fertilizers after 1960. The early release of N was due to plowing of the prairies, with the more recent release due to fertilizer inputs that exceed N in harvested crops. Both greatly increased the riverine export of N to the Mississippi River and the Gulf of Mexico. These patterns of N inputs and outputs were probably similar for all areas of the agricultural Midwest. However, the timing of initial cultivation and drainage varied among states, and generally moved in a westerly direction, which probably moderated impacts at the scale of the Mississippi River Basin. Nonetheless, the flux of N to the Gulf (and possible hypoxia episodes) was probably large in the late 1800s, decreasing to the 1950s, and then increasing again in the 1980s. The variation in riverine transport of N from Illinois has resulted primarily from factors such as tile drainage, which altered the hydrology of the rivers, streams, and wetlands of the state and also from factors such as loading of untreated sewage, which influenced the importance of denitrification.

## ACKNOWLEDGEMENTS

This project was funded in part by the Illinois Council on Food & Agricultural Research, Water Quality Strategic Research Initiative.

## References

1. Carpenter, S.R., Caraco, N.E., Correll, D.L., Howarth, R.W., Sharpley, A.N., and Smith, V.H. (1998) Nonpoint pollution of surface waters with phosphorus and nitrogen. *Ecol. Appl.* **8**, 559–568.
2. Vitousek, P.M., Aber, J.D., Howarth, R.W., Likens, G.E., Matson, P.A., Schindler, D.W., Schlesinger, W.H., and Tilman, D.G. (1997) Human alteration of the global nitrogen cycle: sources and consequences. *Ecol. Appl.* **7**, 737–750.
3. Jordan, T.E. and Weller, D.W. (1996) Human contributions to terrestrial nitrogen flux. *BioScience* **46**, 655–664.
4. Howarth, R.W., Billen, G., Swaney, D., Townsend, A., Jaworski, N., Lajtha, K., Downing, J.A., Elmgren, R., Caraco, N., Jordan, T., Berendse, F., Freney, J., Kudeyarov, V., Murdoch, P., and Zhao-Liang, Z. (1996) Regional nitrogen budgets and riverine N & P fluxes for the drainages to the North Atlantic Ocean: natural and human influences. *Biogeochemistry* **35**, 75–139.
5. Keeney, D.R. (1979) A mass balance of nitrogen in Wisconsin. *Trans. Wisc. Acad. Arts Lett.* **67**, 95–102.
6. David, M.B. and Gentry, L.E. (2000) Anthropogenic inputs of nitrogen and phosphorus and riverine export for Illinois, USA. *J. Environ. Qual.* **29**, 494–508.
7. Jordan, T.E., Correll, D.L., and Weller, D.E. (1997) Effects of agriculture on discharges of nutrients from coastal plain watersheds of Chesapeake Bay. *J. Environ. Qual.* **26**, 836–848.
8. Goolsby, D.A., Battaglin, W.A., Lawrence, G.B., Artz, R.S., Aulenbach, B.T., Hooper, R.P., Keeney, D.R., and Stensland, G.J. (1999) Flux and Sources of Nutrients in the Mississippi-Atchafalaya River Basin. Topic 3. Report of the Integrated Assessment on Hypoxia in the Gulf of Mexico. NOAA Coastal Ocean Program Decision Analysis Series No. 17. NOAA Coastal Ocean Program, Silver Spring, MD.
9. McIsaac, G.F., David, M.B., Gertner, G.Z., and Goolsby, D.A. (2001) Net anthropogenic N inputs to the Mississippi River Basin and nitrate flux to the Gulf of Mexico. *Nature*. in press.
10. Illinois Department of Agriculture. (1988) Illinois Agricultural Statistics Annual Summary – 1988. Bulletin 88-1. Illinois Agricultural Statistics Service, Springfield, Illinois.
11. U.S. Bureau of Census. (1870-1920) 9th through 14th Census of the U.S. U.S. Department of Commerce, Bureau of the Census, Suitland, MD.
12. U.S. Census of Agriculture. (1925-1945) Reports for States with Statistics for Counties and a Summary for the United States. U.S. Department of Commerce, Bureau of Census, Suitland, MD.
13. Clark, C.C. (1908) Number and farm value of farm animals in the United States 1867-1907. U.S. Department of Agriculture Bureau of Statistics Bulletin 64.
14. Cooper, M.R. (1917) Cost of keeping farm horses and cost of horse labor: a study of records for 316 horses on 27 farms in Illinois, Ohio, and New York. U.S. Department of Agriculture Bulletin 560.
15. Johnson, O.R. and Green, R.M. (1918) Cost of horse labor on the farm. University of Missouri Agriculture Experiment Station Bulletin 152. Columbia, MO.
16. Edmonds, J.L. (1934) The farm horse: its feeding, care, and breeding. University of Illinois Agriculture Experiment Station Circular No. 424. Urbana-Champaign, IL.
17. USEPA. (2000) National Air Pollutant Emission Trends, 1900–1998. Available at http://www.epa.gov/ttn/chief/trends/trends98/chapter3.pdf (verified 03 July 2001).
18. DuBois, J.D. and Kapustka, L.A. (1983) Biological nitrogen fixation in an Ohio relict prairie. *Am. J. Bot*. **70**, 8–16.
19. Alexander, J.D. and Darmody, R.G. (1991) Extent and organic matter content soils in Illinois soil associations and counties. Agronomy Special Report 1991-03. University of Illinois, Urbana-Champaign.
20. Jenny, H. (1941) *Factors of Soil Formation*. McGraw Hill, New York.
21. Mann, L.K. (1986) Changes in soil carbon storage after cultivation. *Soil Sci*. **142**, 279–288.
22. Hopkins, C.G. and Pettit, J.H. (1906) The fertility in Illinois soils. Illinois Agricultural Experiment Station Bulletin 123. Urbana-Champaign, IL.
23. Rost, C.G. and Always, F.J. (1921) Minnesota glacial soil studies: I. Comparison of soils on the late Wisconsin and Iowan drifts. *Soil Sci.* **11**, 161–200.

24. Russell, J.C. and McRuer, W.G. (1927) The relation of organic matter and nitrogen content to series and type in virgin grassland soils. *Soil Sci.* **24**, 421–452.
25. Anderson, M.A. (1949) Some Physical and Chemical Properties of Six Virgin and Six Cultivated Iowa Soils [Thesis]. Iowa State College (now Iowa State University), Ames.
26. Thompson, L.M., Black, C.A., and Zoellner, J.A. (1954) Occurrence and mineralization of organic phosphorus in soils with particular reference to associations with nitrogen, carbon and pH. *Soil Sci*. **77**, 185–196.
27. Bogue, M.B. (1951) The Swamp Land Act and Wet Land Utilization in Illinois, 1850-1890. *Agric. Hist.* **25**, 159–180.
28. Tilman, D., Fargione, J., Wolff, B., D'Antonio, C., Dobson, A., Howarth, R., Schindler, D., Schlesinger, W.H., Simberloff, D., and Swackhamer, D. (2001) Forecasting agriculturally driven global environmental change. *Science* **292**, 281–284.
29. Conservation Technology Information Center. (2001) National Crop Residue Management Survey. http://www.ctic.purdue.edu/CTIC/CTIC.html
30. Needleman, B.A., Wander, M.M., Bolero, G.A., Boast, C.W., Sims, G.K., and Bullock, D.G. (1999) Interaction of tillage and soil texture: biologically active soil organic matter in Illinois. *Soil Sci. Soc. Am. J.* **63**, 1326–1334.
31. Wander, M.M., Bidart, M.G., and Aref, A. (1998) Tillage impacts on depth distribution of total and particulate organic matter in three Illinois soils. *Soil Sci. Soc. Am. J.* **62**, 1704–1711.
32. Yang, X. and Wander, M.M. (1999) Tillage effects on soil organic carbon distribution and storage in a silt loam soil in Illinois. *Soil Tillage Res.* **52,** 1–9.
33. Fehrenbacher, J.B. (1973) Loess stratigraphy, distribution, and time of deposition in Illinois. *Soil Sci*. **115**, 176–182.
34. Palmer, A.W. (1903) Chemical Survey of the Waters of Illinois Report for the Years 1897-1902. University of Illinois.
35. Kofoid, C.A. (1903) Plankton studies IV. Plankton of the Illinois River 1894-1899, with Introductory Notes of the Hydrography of the Illinois River and its Basin. Part I. Quantitative Investigations and General Results. *Bull. Ill. State Lab. Nat. Hist.* **6**, 95–635.
36. Junk, W.J., Bayley, P.B., and Sparks, R.E. (1989) The flood pulse concept in river-floodplain systems. In Proceedings of the International Large River Symposium. Dodge, D.P., Ed. *Can. Spec. Pub. Fish. Aquat. Sci.* **106,** 110–127.
37. David, M.B., Gentry, L.E., Kovacic, D.A., and Smith, K.M. (1997) Nitrogen balance in and export from an agricultural watershed. *J. Environ. Qual*. **26**, 1038–1048.

| | | |
|---|---|---|
| Received: | July | 6, 2001 |
| Revised: | October | 3, 2001 |
| Accepted: | October | 5, 2001 |
| Published: | October | 23, 2001 |
